# Discovery, characterization and potential roles of a novel NF-YA*x* splice variant in human neuroblastoma

**DOI:** 10.1186/s13046-019-1481-8

**Published:** 2019-12-05

**Authors:** Lucia Cappabianca, Antonietta Rosella Farina, Lucia Di Marcotullio, Paola Infante, Daniele De Simone, Michela Sebastiano, Andrew Reay Mackay

**Affiliations:** 1 Department of Applied Clinical and Biotechnological Sciences, University of L’Aquila, Via Vetoio, Coppito 2, 67100 L’Aquila, Italy; 2 Department of Molecular Medicine, La Sapienza University of Rome, 00161 Rome, Italy; 3 Istituto Pasteur-Fondazione Cenci Bolognetti, La Sapienza University of Rome, 00161 Rome, Italy; 4 Center for Life Nanoscience @ Sapienza, Istituto Italiano di Tecnologia, 00161 Rome, Italy

**Keywords:** NF-Y, NF-YA, Neuroblastoma, Alternative splicing, Necroptosis, KIF1Bβ, Cancer stem cells, Genotoxic stress

## Abstract

**Background:**

Identification of novel cancer-associated splice variants is of potential diagnostic, prognostic and therapeutic importance. NF-Y transcription factor is comprised of NF-YA, NF-YB and NF-YC subunits, binds inverted CCAAT-boxes in ≈70% of gene promoters, regulates > 1000 cancer-associated genes and proteins involved in proliferation, staminality, differentiation, apoptosis, metabolism and is subject to component alternative splicing. RT-PCR evaluation of alternative NF-YA splicing in primary human neuroblastomas (NBs), led to discovery of a novel NF-YA*x* splice variant*,* also expressed during mouse embryo development and induced by doxorubicin in NB cells. Here, we report the discovery and characterisation of NF-YA*x* and discus its potential roles in NB.

**Methods:**

NF-YA*x* cDNA was RT-PCR-cloned from a stage 3 NB (provided by the Italian Association of Haematology and Paediatric Oncology, Genova, IT), sequenced and expressed as a protein using standard methods and compared to known fully-spliced NF-YA*l* and exon B-skipped NF-YA*s* isoforms in: EMSAs for capacity to form NF-Y complexes; by co-transfection, co-immunoprecipitation and Western blotting for capacity to bind Sp1; by IF for localisation; in AO/EtBr cell-death and colony formation assays for relative cytotoxicity, and by siRNA knockdown, use of inhibitors and Western blotting for potential mechanisms of action. Stable SH-SY5Y transfectants of all three NF-YA isoforms were also propagated and compared by RT-PCR and Western blotting for differences in cell-death and stem cell (SC)-associated gene expression, in cell-death assays for sensitivity to doxorubicin and in in vitro proliferation, substrate-independent growth and in vivo tumour xenograft assays for differences in growth and tumourigenic capacity.

**Results:**

NF-YA*x* was characterized as a novel variant with NF-YA exons B, D and partial F skipping, detected in 20% of NF-YA positive NBs, was the exclusive isoform in a stage 3 NB, expressed in mouse stage E11.5–14 embryos and induced by doxorubicin in SH-SY5Y NB cells. The NF-YA*x* protein exhibited nuclear localisation, competed with other isoforms in CCAAT box-binding NF-Y complexes but, in contrast to other isoforms, did not bind Sp1. NF-YA*x* expression in neural-related progenitor and NB cells repressed Bmi1 expression, induced KIF1Bβ expression and promoted KIF1Bβ-dependent necroptosis but in NB cells also selected tumourigenic, doxorubicin-resistant, CSC-like sub-populations, resistant to NF-YA*x* cytotoxicity.

**Conclusions:**

The discovery of NF-YA*x* in NBs, its expression in mouse embryos and induction by doxorubicin in NB cells, unveils a novel NF-YA splice mechanism and variant, regulated by and involved in development, genotoxic-stress and NB. NF-YA*x* substitution of other isoforms in NF-Y complexes and loss of capacity to bind Sp1, characterises this novel isoform as a functional modifier of NF-Y and its promotion of KIF1Bβ-dependent neural-lineage progenitor and NB cell necroptosis, association with doxorubicin-induced necroptosis and expression in mouse embryos coinciding with KIF1Bβ-dependent sympathetic neuroblast-culling, confirm a cytotoxic function and potential role in suppressing NB initiation. On the other hand, the in vitro selection of CSC-like NB subpopulations resistant to NF-YA*x* cytotoxicity not only helps to explain high-level exclusive NF-YA*x* expression in a stage 3 NB but also supports a role for NF-YA*x* in disease progression and identifies a potential doxorubicin-inducible mechanism for post-therapeutic relapse.

## Background

Alternative gene splicing is a fundamental physiological mechanism for the differential expression of proteins from the same gene coding sequence and is largely responsible for the increased proteomic complexity of higher organisms that cannot be explained by differences in individual gene numbers alone [1]. Aberrant alternative splicing has been reported in cancer and is promoted by stress within the tumour microenvironment, oncogenic viral infection, gene translocation and oncogene de-regulation of splice factor expression. Cancer-associated alternative splicing has been shown to inactivate onco-suppressors and activate oncogenes, making the identification of novel cancer-associated splice isoforms of potential diagnostic, prognostic and therapeutic importance [1–5].

The ubiquitous transcription factor NF-Y binds inverted CCAAT-boxes (5′-ATTGG-3′) in ≈70% of gene promoters, recruits other transcription factors and proteins to promoters and regulates > 1000 cancer-associated genes involved in proliferation, stemness, differentiation, apoptosis, miRNA expression and metabolism [6–9]. NF-Y also regulates chromatin de-condensation during mitotic bookmarking and post-mitotic transcriptional re-activation, and acts as a bi-directional histone-like transcription factor, switching promoter histone methyl-marks from positive to negative. NF-Y is necessary for development, its inactivation is embryonically lethal and alterations in NF-Y activity have wide-ranging effects on cell behaviour [6–10]. The involvement of NF-Y in cancer is underpinned by its role in proliferation, the presence of inverted CCAAT-boxes in the promoters of many cancer-associated genes and interaction with and regulation of cancer-related proteins, including c-fos, wild type and mutant p53, p73, ΔNp63α, p21, Ash2L, BRCA-1, cMyc, Sox9, lamin, ZHX1/2 and in particular the ubiquitous cancer-related transcription factor Sp1 [6–11].

NF-Y is a hetero-trimer composed of NF-YA, NF-YB and NF-YC subunits, all of which are required for DNA binding and transcriptional activity. NF-YB complexes with NF-YC prior to binding NF-YA, which confers DNA binding and transcriptional activity to the NF-Y complex [6–8, 12–14]. The 29.44 kb *NF-YA* gene localises to chromosome 6p21, is organized into 9 exons [15] and is predominantly expressed as a fully-spliced 42 kDa, 347 amino acid (aa) long-form NF-YA*l* with glutamine-rich, S/T-rich transactivation, subunit-interaction and DNA-binding domains or an alternative exon B-spliced 40 kDa, 318 aa short-form NF-YA*s*, deleted of glutamine-rich aa’s 26–54 [15, 16]. Minor NF-YA isoforms also include a 3 bp aa 27 deletion-variant, a 18 bp aa 548–565 deletion-variant and L2-L6 alternative splice variants deleted in glutamine-rich and S/T domains, driven by nucleotide 79, 154 and 548 deletions at A/B, B/C and E/F splice junctions [15, 17].

Alternative splicing of the *NF-YA* gene has been implicated in the regulation of cell staminality, differentiation, apoptosis and transformation. NF-YA*s* forms part of the stem cell (SC) transcriptional circuitry, predominates in embryonic SCs and is lost upon SC differentiation. In contrast, NF-YA*l* promotes differentiation and loss of NF-YA expression induces senescence or apoptosis. Alternative NF-YA*s* splicing is promoted by the oncogenic polyomavirus SV40 and by *v-ras* oncogene and converts tumor-suppressing, differentiation-promoting NF-Y complexes predominated by NF-YA*l* into tumor and CSC promoting complexes predominated by NF-YA*s* [8, 18–23].

Neuroblastomas (NB) are aggressive embryonic tumours of neural crest origin, derived from immature sympathetic neuroblasts [24]. These primitive tumours initiate under conditions that impair sympathetic neuroblast culling during development, reported to depend upon either loss of the *KIF1B* gene associated with chromosome 1p36-deletion, germline KIF1B mutations or Nmyc amplification [25–33]. NF-Y involvement in NB pathogenesis and progression, however, has received scant attention. In the few existing reports, NF-Y has been shown to be critical for expression of soluble guanyl cyclase in NB cells required for cGMP production and differentiation [34] and is involved in elevated glypican 3 expression in NBs [35]. NF-Y and Sp1 transcription factors combine to promote tetramethylpyrazine-induced neuronal differentiation of NB cells [36] and regulate expression of the α3 Na+, K + -ATPase subunit, essential for maintaining electrochemical gradients across cell membranes [37]. Suboptimal NF-Y function in NB cells has also been implicated in de-regulating the matrix metalloproteinase and tissue inhibitor of metalloproteinase equilibrium, resulting in invasion [38] and increased expression of the NF-YA subunit has been reported to differentiate between aggressive stage 4 NBs and stage 4S NBs that exhibit spontaneous regression [39].

Considering the relative absence of studies of NF-Y expression in NB, combined with reports associating fully spliced NF-YA*l* with cellular differentiation and reduced malignancy and associating alternative exon B spliced NF-YA*s* with cellular staminality and increased malignancy [18–23], we initiated a study of NF-YA*l* and NF-YA*s* expression in human primary NBs. This led to the unexpected discovery of a novel NF-YA*x* splice variant, with NF-Y functional modifying activity, which forms the subject of this report.

## Materials and methods

### Aim, design and setting

The aim of this study was to report the discovery and characterization of the novel alternative NF-YA*x* splice variant, discovered as the exclusive NF-YA isoform expressed in an advanced stage 3 NB, expressed during mouse embryo development and induced in NB cells by doxorubicin, and to provide insights into its potential function in NB pathogenesis and progression. Experimental design included cloning and sequence characterization, expression vector construction, protein expression and characterization, transient and stable transfection and biological characterizations in terms of growth, cytotoxicity and tumorigenicity.

### Characteristics of participants and materials

RNAs from primary human stage 1, 2, 3 and 4 NBs (International NB Staging System) were kindly provided by the tissue bank of the Italian Association of Haematology and Pediatric Oncology (Genova, Italy). Patient age, sex or gender was not provided. RNAs from stage E8.5-E18.5 mouse embryos were provided by Dr. Rita Gallo (University of L’Aquila, L’Aquila, Italy). The following cell lines were used: stable transfected pcDNA3.1 control, TrkAIII and TrkT3 SH-SY5Y cell lines [3]; SV40 large T-antigen immortalized ST14A rat neuronal progenitor cells (kindly provided by Dr. Elena Cattaneo) [40]; NB cell lines SK-N-SH (HTB-11); SH-SY5Y (CRL-2266); IMR32 (CCL-127); SK-N-BE (CRL-2271); LAN-1 (HB-8568), HEK-293 embryonic neural lineage kidney cells [41, 42] (CRL-1573) and SL2 Sneider’s Drosphila Line 2 insect (CRL-1963) cell lines from ATCC (Rockville, MD); LAN-5 NB cell line from DSMZ (ACC673) (Braunschweig, Germany), and SMS-KCNR and SH-EP NB cell lines from Dr. U.P. Thorgiersson (NCI, NIH, Bethesda, MD). Cells were routinely grown at 37 °C and 5% CO_2_, in ATCC or DSMZ recommended culture medium (RPMI, DMEM), supplemented with appropriate antibiotics (Zeocin for stable-transfectants, penicillin and streptomycin) and 10% foetal calf serum. SL2 cells were cultured in Schneider’s Drosophila medium supplemented with 10% foetal bovine serum. Human foetal brain-derived neural stem cells were maintained as neurospheres in NPMM maintenance medium supplemented with Growth and Supplement Singlequotes, as directed (Cambrex Bio Science, Milan, Italy). All cell lines were authenticated at source. The following antibodies were used: KIF1Bβ (A301-055A-M, Bethyl Laboratories, Montgomery, TX); EglN3 (BS0532R-TR, Bioss, Woburn, MA); β-Actin (sc-47,778), Caspase 9 (sc-819), Mcl1 (sc56077), Bcl2 (sc-509), NF-YA (sc-10,779), NF-YB (sc-13,045), Sp1 (sc-420), Ubiquitinated proteins (sc-9133, P62 (sc48402), Bcl-xL (sc-136,207) (Santa Cruz, Dallas, TX); Alix (92880), Caspase 3 (9662), PARP (9642), pJNK (4668) and JNK (9258) (Cell Signaling Technology, Danvers, MA).

### RNA purification and RT-PCR

Total RNAs from cell lines and xenograft tumor tissues were purified using an RNA-easy Plus RNA purification kit, as directed (Qiagen, Milan, Italy). All RNAs (1 μg) were reverse transcribed using a Maxima H Minus First Strand cDNA synthesis kit, as directed (Thermo-Fisher Scientific, Waltham, MA) and subjected to semi-quantitative PCR (35 cycles of 40 s at 95 °C, 30 s at 1° below melting temperature and 42 s at 72 °C), using the following primer sets: human NF-YA (5′-AATAGTTCGACAGAGCAGATTG-3′ and 5′-TCCTGCCAAAC TGGCTGCTGGGAT-3′, generating products of 575 bp for NF-YA*l,* 488 bp for NF-YA*s* and 338 bp for NF-YA*x*); mouse NF-YA (5′-AAACAGCAATAGTTCCACAGAGCAGATCG-3′ and 5′-CGATCTGCTCTGTGGAACTATTGCTG TT T-3′; products of 582 bp for NF-YA*l*, 494 bp for NF-YA*s* and 361 bp for NF-YA*x*); CD133 (5′-GCTGATGTTGAAACTGCTTGAG-3′ and 5′-GGTGCCGTTGCCTTGG-3′); SOX2 (5′-CCAAGACGCTCATGAAGAAG-3′ and 5′-TGGTCATGGAGTTGTACTGC-3′;); CD117 (5′-AACGCTCGAGTACCTGTGAA-3′ and 5′-GACAGAATTGATCCGCACAG-3′); Nestin (5′-GGATCAGATGACATTAAGACCC-3′ and 5′-TCCAGTGGTTCTTGAATTTCC-3′); Nanog (5′-TCTCTCCTCTTCCTTCCTCCATG-3′ and 5′-CTGTTTGTAGCTGAGGTTCAGGATG-3′); p75^NGFR^ (5′-GTGGGACAGAGTCTGGGTGT-3′ and 5′-AAGGAGGGGAGGTGATAGGA-3′); Bmi1 (5′-GCTGCCAATGGCTCTAATGA-3′ and 5′-TGCTGGGCATCGTAAGTATCTT-3′); Bcl2 (5′-GACT TCGCCGAGATGTCCAGC-3′ and 5′-CAAGCT CCCACCAGGGCCAAAC-3′); Mcl1 (5′-AAGCCAATGGGCAGGTCT-3′ and 5′-TGTCCAGTTTCCGAAGCAT-3′); BclxL (5′-GAACGGCGGCTGGGATACTTTTG-3′ and 5′-GT GAATTCTGAGGCCAAGGGAAC-3′); PUMA (5′-GCATGGGGTCTGCCCAGG-3′ and 5′-CCGCCGCTCGTACTGTGC-3′); BAD (5′-CAGTCACCAGCAGGAGCAG-3′ and 5′-CCCTCCCTCCAAAGGAGACAG-3′); BAX (5′-ATGGACGGGTCCGGGGAGCAG-3′ and 5′-GCACCAGTTTGCTGGCAAAGTAG-3′); EglN3 (5′-GCGTCTCCAAGCGACA-3′ and 5′-GTCTTCAGTGAGGGCAGA-3′); KIF1Bβ (5′-CAGTGACTGTAAGTTGTCTGATATA-3′ and 5′-GTAAAGAGGCTCCTTGAAAT-3′); GAPDH (5′-CTGCACCACCAACTGCTTAG-3′ and 5′-AGGTCCACCACTGACACGTT-3′) and 18S rRNA (5′-AAACGGCTACCA CATCCAAG-3′ and 5′-CGCTCCCAAGATCCAACTAC-3′).

### NF-YA variant cDNA cloning and sub-cloning

NF-YA*x* coding cDNA was cloned from the primary human stage 3 NB, exhibiting exclusive NF-YA*x* expression. NF-YA*l,* NF-YA*s,* NF-YB and NF-YC cDNAs were cloned from SH-SY5Y mRNA, using primers 5′-CCTCCTGATTGGGTTTCGGAGT-3′ and 5′-GGGGTTAGGACACTCGGATGAT-3′ spanning fully-spliced human NF-YA*l* cDNA (NM_002505.5); primers 5′-GGTTCTGACAGTATTTCATGACAA-3′ and 5′-CAGATCATGAAAACTGAATTTGCT-3′ spanning fully spliced human NF-YB cDNA (NM_006166) and primers 5′-GGACTCCTGAGCACAGTTGTCGAG-3′ and 5′-CCTTGGCCTTGCCAGCTCAGGCCC-3′ spanning fully spliced NF-YC cDNA (HSU78774). RT-PCR products were agarose gel purified, sub-cloned into the TA cloning vectors (Invitrogen, Thermo-Fisher Scientific, Waltham, MA) and sequenced in an ABI prism automated DNA-sequencer. Full length NF-YA*l,* NF-YA*s,* NF-YA*x*, NF-YB and NF-YC cDNA clones were then sub-cloned into mammalian pcDNA3.1Zeo (+) and pAc insect expression vectors (Thermo-Fisher Scientific, Waltham, MA), together with cDNA for full-length Sp1, available in our laboratory. NF-YA*dn* expression vector was from Dr. R. Mantovani (University of Milan, Italy).

### SiRNA knockdown

SiRNA knockdown was achieved using a TriFECTa Dicer-Substrate RNAi kit and three KIF1B-specific Dicer-Substrate siRNA duplexes (hs.Ri.KIF1B.13.1: 5′-UCCACUGAGAAGGUCAGUAAAAUCA-3′ and 5′-UGAUUUUACUGACCUUCUCAGUGGAAA-3′; hs.Ri.KIF1B.13.2: 5′-CAAGGAAUCCAAAUGCAUCAUUCAG-3′ and 5′-CUGAAUGAUGCAUUUGGAUUCCUUGCU-3′; hs.Ri.KIF1B.13.3: 5′-AGCUAUUGAACGUUUAAAG GAAUCA-3′), as directed (Integrated DNA Technologies, Bologna, Italy). Briefly, 1 × 10^5^ cells/ml HEK-293 cells on 24 well plates were grown overnight to ≈ 80% confluence and co-transfected with either 50 nM of negative control siRNA duplex (5′-CGUUAAUCGCGUAUAAUACGCGUAT-3′ and 5′-AUACGCGUAUUAUACGCGAUUAACGA C-3′) or 50 nM of a mix of KIF1B-specific siRNA duplexes, plus 1 μg/ml of pcDNA3.1 NF-YA*x* expression plasmid or empty pcDNA control plasmid, using TransIT-X2 Dynamic Delivery System, as directed (Mirus Bio, www.mirusbio.com/6000). Sham-transfected controls received transfection reagent alone. Knockdown of NF-YAx-induced KIF1Bβ expression was confirmed at 24 h by RT-PCR and the effect of knockdown assessed on NF-YA*x*-induced cell death at 48 h, by phase contrast and AO/EtBr cell death assays. Knockdown experiments were performed in duplicate and repeated (*n* = 4). Transfection efficiency was confirmed using HPRT-S1 DS positive control and validated using a negative control duplex (NC1), as directed (Integrated DNA Technologies, Bologna, Italy).

### Immunoprecipitations and Western blotting

Cell proteins, extracted in lysis buffer (PBS containing 0.5% sodium deoxycholate, 1% NP40, 0.1% SDS, 1 mM sodium orthovanadate, 1 mM PMSF, 1 μg/ml of pepstatin A and Aprotinin), were analysed by reducing SDS-PAGE/Western blotting, as previously described [3]. For immunoprecipitations, cell extracts (200–500 μg), pre-cleared with IgG and Protein A Sepharose (Fast flow, Sigma-Aldrich, St Louis, MI), were incubated overnight with primary antibody (0.1–1.0 μg), with rotation at 4 °C, then 20 μl of Protein A Sepharose in lysis buffer was added and incubated for 30 min at 4 °C. Protein A Sepharose/IgG conjugates, collected by centrifugation (10,000 x g for 5 min), were washed in lysis buffer and examined by reducing SDS-PAGE/Western blotting.

### Stable SH-SY5Y transfection

SH-SY5Y cells were stable-transfected with pcDNA 3.1 NF-YA*l*, NF-YA*s* or NF-YA*x* or empty pcDNA3.1Zeo expression vectors, as previously described [3]. Zeocin-resistant colonies were isolated upon appearance, clonally expanded and characterized by RT-PCR and Western blot.

### Transient transfections

Cells at 1 × 10^5^/ml were grown overnight to ≈80% confluence on 6 or 24 well plates, transfected with NF-YA variant or empty plasmid DNA (1 μg/ml) in Fugene HD, as directed (Promega, Madison, WI), washed at 6 h, grown in complete medium, monitored at 6, 12, 24 and 48 h for evidence of cytotoxicity and photographed. At 48 h, suspension and adherent cell populations were separated and either analysed in AO/EtBr cell-death assay, used for RNA purification and RT-PCR analysis or extracted for Western blotting. Transfection efficiency was estimated using pEGFP-N1 green fluorescent protein reporter plasmid (Clontech, Mountain View, CA).

### Southern blotting

Southern blots of NF-YA and GAPDH RT-PCR products were prepared, as previously described [3]. Briefly, RT-PCR products from primary human NB RNAs (1 μg) and doxorubicin-treated SH-SY5Y mRNAs (100 ng), resolved by 1.5% agarose gel electrophoresis, were in-gel denatured with 0.5 M NaOH, transferred to Hybond N+ nylon membranes (Amersham International, Little Chalfont, UK) and hybridized with P^32^-labelled probes for either NF-YA exon C (5′-CAAGGGCAGCCATTAATGGTGCAGGTCAGTGGAGGCCAGCTAATCACATCAACTGGCCAACCCATCATG-3′), NF-YA exon D (5′-AGGGCCAGCAGGGCCAGACCCAGCAGATCATCATCCAGCAGCCCCAGACGGCT GTCACTGCTGGCCAGA-3′) or GAPDH and visualized by autoradiography.

### MTS proliferation and thymidine incorporation assays

In MTS proliferation assays (Promega, Madison, WI), 5 × 10^3^ cells in 96-well microtiter plates were grown for 0–4 days. MTS reagent (20 μl) was added at 24-h intervals to individual wells and colorimetric conversion monitored at 492 nm every 30 min for 4 h in a microtiter plate reader. Assays were performed in duplicate and repeated three times (*n* = 6). For thymidine incorporation assays, 5 × 10^3^ cells seeded on 96-well microtiter plates were grown for 0–4 days. At 24-h intervals, 1 μCi of tritiated [^3^H] thymidine (Amersham International, Little Chalfont, UK) was added and incubated for 4 h. Cultures were then washed in pre-warmed PBS, trypsinized, vacuum transferred onto 3MM Whatman paper filters and counted in a β-Scintillation counter (Becton, Dickinson and Company, Franklyn Lakes, NJ). Assays were performed in duplicate and repeated 3 times (n = 6).

### In vitro protein translation

NF-YA*l,* NF-YA*s,* NF-YA*x,* NF-YB and NF-YC proteins were translated in vitro from linearized cDNAs (1 μg), in the presence of ^35^S labelled methionine, for 90 min at 37 °C, in a rabbit reticulocyte T_N_T T7 Quick Coupled Transcription/translation System, as directed (Promega, Madison, WI). Translated proteins were resolved by reducing SDS-PAGE autoradiography. For EMSAs, ^35^S-labelled methionine was omitted from in vitro translation reactions.

### In vitro Colony formation assays

Cell suspensions (1 × 10^5^/ml) in 6 well culture plates were grown overnight to ≈80% confluence, transfected with empty pcDNA3.1 or pcDNA3.1 NF-*YAl,* NF-*YAs* or NF-YA*x* expression vectors (2 μg/ml) in Fugene, as directed (Promega, Madison, WI), and grown for a further 48 h. Zeocin (200 μg/ml) was then added, replaced every 3 days and colonies grown for 21 days, fixed in 100% Methanol, stained in 0.5% w/v crystal violet, 1% Formaldehyde, 1x PBS, 1% methanol, washed in tap water, air dried, photographed and counted. Assays were performed in triplicate and repeated 3 times (*n* = 9).

### Neural stem cell sphere growth assays

Cells seeded at 1 × 10^5^/ml in vertical T75 culture flasks (NUNC) were grown in DMEM/F1, containing 0.6% glucose, 1 x B27 supplement (Gibco, Thermo-Fisher Scientific, Waltham, MA), 1 x N2 supplement (Gibco), antibiotics (1 x Pen/Strep), glutamine, EGF (40 ng/ml) and FGF (40 ng/ml), and monitored for formation of neuro-spheres consisting of > 50 cells. Assays were performed in duplicate and repeated (*n* = 4).

### Direct RT-PCR sequencing

Agarose gel-purified RT-PCR product sequencing was performed using BigDye Direct Cycle Sequencing kit, as directed (Thermo-Fisher Scientific, Waltham, MA). Briefly, cDNAs (final concentration 4 ng/ml) were added to reaction mixtures containing forward or reverse primer (0.8 μM), BigDye direct PCR master mix, deionized water in a final volume of 20 μl and subjected to 35 PCR cycles (94 °C and for 10 min and 96 °C for 10 s, 55 °C for 5 min and 60 °C for 4 min). PCR reactions were mixed with 2 ul of NaAc and 50 μl absolute ethanol, incubated for 10 mins, spin-dried at 120000 rpm for 20 mins and sequenced in a mono-capillary sequencer, as directed (ABI PRISM 310, Life Technologies, Monza, IT).

### Tumor growth in soft agar

Single-cell suspensions (passed through a gauge × 18 syringe needle) of 5 × 10^4^ cells were mixed in a 33% solution of agar (BiTec; Difco) in RPMI containing 5% FCS at 37 °C and layered onto a solid 0.6% agarose substrate prepared in the same growth medium. Following agar solidification, complete medium was added, replaced every 2 days and growth monitored over 14 days. Assays were performed in duplicate and repeated (*n* = 4).

### Tumor growth in NGS mice

Tumorigenesis in vivo was performed as previously described [3]. Stable-transfected SH-SY5Y cells, prepared as a single-cell suspension in PBS without Ca^2+^ and Mg^2+^, were injected subcutaneously into the flanks of anesthetized, 6-week-old female NGS mice (Charles River, Calco, Italy), at a concentration of 1 × 10^7^ cells in 200 μl per site. Tumor initiation was recorded at a minimum volume (tumor length × [tumor width]^2^ × 0.44) of 12 mm^3^ and animals sacrificed at 21 days. Groups consisted of 5 animals and the assay performed once (*n* = 5). All experiments were performed in accordance with Italian national and Rome University guidelines.

### Nuclear extracts and EMSA

Nuclear extracts and EMSAs were performed as previously described [3]. Briefly, binding reactions, performed at room temperature for 20 min, contained ^32^P-5′ end-labelled, double-stranded oligonucleotide probes, 2 μg of poly-deoxyinosinic-deoxy-cytidilic acid, 5 μg of nuclear extract and additional competitor DNAs or antibodies as specified in the figure legends. The oligonucleotides used were as follows: inverted CCAAT-box: 5′-GGGAGACCGTACGTGATTGGTTAATCTCTT-3′ and non-specific: 5′-GGTCGATAGGGAATTTACACGC-3′ oligonucleotides. All oligonucleotides were double stranded, the complementary affinity strands are not indicated. Assays were repeated 3 times (*n* = 3).

### Cell death assays

Cell death assays were as previously described [43]. Suspension or adherent cells, detached in ice cold PBS containing 1 mM EDTA, were transferred into sterile tubes, pelleted at 1000 x g at 4 °C, washed in ice cold PBS, re-pelleted, re-suspended in 25 μl of PBS containing 2 μl of acridine orange/ethidium bromide solution (100 μg/ml acridine orange and 100 μg/ml ethidium bromide in PBS), mounted onto glass slides, examined immediately under a Zeiss “Axioplan-2” fluorescence microscope, digitally photographed and dead (orange/red nuclei) and live cells (green nuclei) counted. Assays were performed in duplicate and repeated 3 times (*n* = 6).

### Indirect immunofluorescence

Cells grown on Nunc glass chamber slides (Sigma-Aldrich, St Louis, MI) were washed in PBS, fixed and permeabilized in 100% ice cold methanol (− 20 °C), incubated for 1 h in blocking solution (1% bovine serum albumin in PBS-0.03% TX100), incubated for 2 h with primary antibody diluted in blocking solution at room temperature, washed, incubated with secondary fluorochrome-conjugated antibody diluted in blocking solution, for 1 h at room temperature, mounted with VectaMount (Vector Laboratories, Berlingame, CA) and observed using a Zeiss Axioplan 2 fluorescence microscope with digital camera and Leica M500 Image Manager software.

### Statistical analysis

Data were analysed by Student’s t-test (https://www.graphpad.com/quickcalcs/ttest1.cfm) and statistical significance associated with probabilities of ≤0.05.

## Results

This study, originally designed to evaluate fully-spliced NF-YA*l* and alternatively exon B spliced NF-YA*s* mRNA expression in primary human NBs, resulted in the unexpected discovery of a novel NF-YA*x* splice variant.

### NF-YA*x* cloning and sequencing

**Fig. 1:**
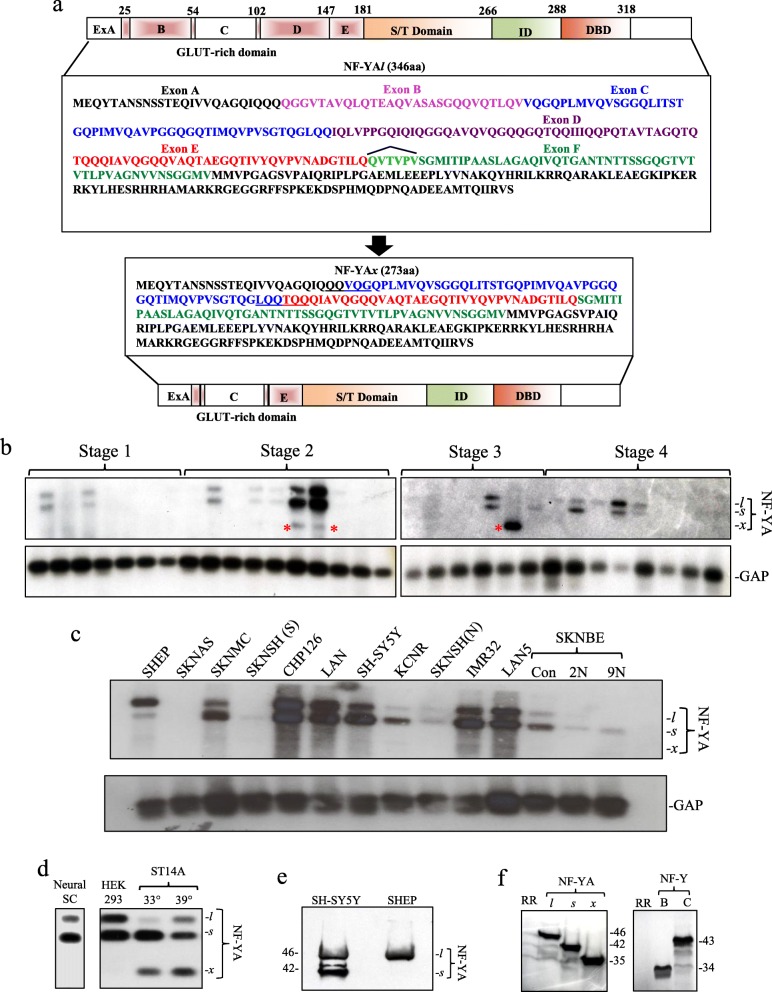
Neuroblastomas express NF-YA*x.* **a** Illustration of NF-YA glutamine-rich transactivation, serine threonine-rich (S/T), interaction (ID) and DNA binding (DBD) domains with relative positions and amino acid sequences of exons B and D, in NF-YA*l* and NF-YA*x* isoforms. Southern blots showing NF-YA isoform and GAP RT-PCR levels in: **b** primary stage 1–4 human NBs; **c** NB cell lines and human embryonic neural SC (left panel) and **d** HEK-293 and ST14A cells (right panel), hybridized with NF-YA exon C (upper panels) and GAP (lower panels) probes (* indicates NF-YA*x*). **e** Western blot showing NF-YA*l* and NF-YA*s* protein levels in SH-SY5Y and SHEP extracts (30 μg). **f** Autoradiographs demonstrating in vitro translated NF-YA*l*, NF-YA*s,* NF-YA*x* (left panel), NF-YB and NF-YC proteins (right panel)

NF-YA*l* (1284 bp) and NF-YA*s* (1197 bp) coding cDNAs from SH-SY5Y cells and full-length NF-YA*x* cDNA (947 bp) from a stage 3 NB were sequenced, and NF-YA*x* characterized as a novel splice variant exhibiting in-frame exons B (87 bp, amino acids (aa) 26–54), D (132 bp, aa 103–146) and partial F (18 bp, aa 182–188) skipping (Fig. 1a) [15].

### NF-YA*x* is expressed in NBs and mouse embryos

RT-PCR of NB RNAs using primers spanning NF-YA exons A-F, generated 575 bp (NF-YA*l*), 488 bp (NF-YA*s*) and 338 bp (NF-YA*x*) products that hybridized to NF-YA exon C probe in Southern blots (Fig. 1b). The 338 bp product, characterized as NF-YA*x* by direct PCR sequencing (not shown), was the exclusive isoform expressed in a stage 3 NB and a minor isoform, together with NF-YA*l* and NF-YA*s,* in two stage 2 NBs but was not detected in either stage 1 or 4 NBs. RNAs from SK-N-MC, SK-N-SH, SH-SY5Y, KCNR, IMR-32 and SK-N-BE NB cell lines, HEK293 embryonic neuronal-lineage kidney cells and human neonatal brain stem cells (SCs) exhibited predominant NF-YA*s* but not constitutive NF-YA*x* expression; CHP-126, LAN-1 and LAN-5 NB cell lines exhibited equivalent NF-YA*s* and NF-YA*l* but not NF-YA*x* expression and SHEP NB cells exhibited predominant NF-YA*l* but not NF-YA*x* expression. Predominant NF-YA*s* with minor NF-YA*x* expression characterized ST14A embryonic striatal neuronal progenitors under progenitor-maintaining (33 °C) and differentiation-inducing (39 °C) conditions [40] (Fig. 1c and d). Western blots confirmed predominant NF-YA*s* protein expression in SH-SY5Y cells, predominant NF-YA*l* expression in SHEP cell extracts (Fig. 1e). NF-YA isoforms translated in vitro were characterized by SDS-PAGE with approximate molecular masses of 46 kDa (NF-YA*l*), 42 kDa (NF-YA*s*) and 35 kDa (NF-YA*x*) (Fig. 1f). Samples were not available for NF-YA*x* protein analysis in primary NBs.

**Fig. 2:**
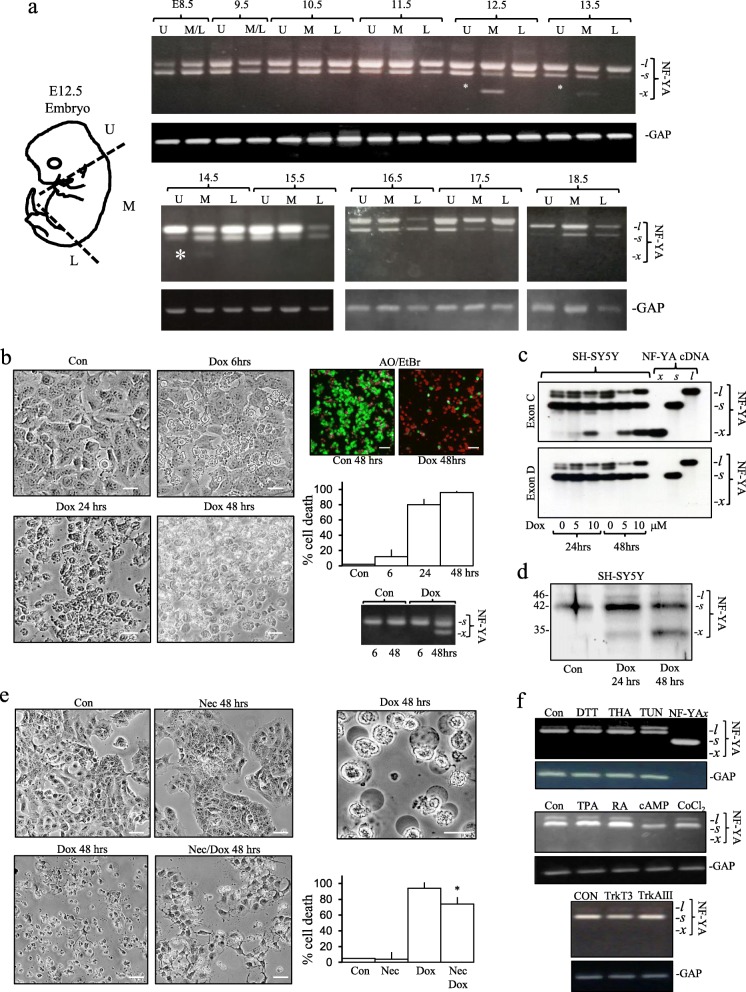
NF-YA*x* is development-regulated and induced by doxorubicin in neuroblastoma cells. **a** Mouse embryo upper (U), mid (M) and lower (L) regions used for RT PCR plus representative agarose gels demonstrating NF-YA isoform (upper panels) and GAP (lower panels) RT-PCR levels in stage E8.5 - E18.5 mouse embryo U, M and L regions (* indicates NF-YA*x*). **b** Representative micrographs (bar = 50 μm) of SH-SY5Y death induced by doxorubicin (10 μM) at 6, 24 and 48 h, a representative AO/EtBr assay demonstrating dead (red) and living (green) SH-SY5Y cells following incubation without (Con 48 h) or with 10 μM doxorubicin (Dox 48 h) (bar = 100 μm) plus a histogram demonstrating time-dependent SH-SY5Y cell death induced by 10 μM doxorubicin at 6, 24 and 48 h, displayed as mean (± SD) percent death in three independent assays, performed in duplicate, and RT-PCR demonstrating the induction of NF-YA*x* expression in SH-SY5Y cells, following 48 treatment with 10 μM doxorubicin (Dox) compared to untreated controls (Con). **c** RT-PCR Southern blots demonstrating time and concentration-dependent doxorubicin induction of NF-YA*x* expression in SH-SY5Y cells together with NF-YA isoform cDNA standards (NF-YA cDNA), in blots hybridized with NF-YA exon C probe (Exon C, upper panel) but not with NF-YA exon D probe (Exon D, lower panel). **d** Western blot demonstrating doxorubicin (10 μM for 24 and 48 h) induction of endogenous NF-YA*x* protein expression in SH-SY5Y nuclear extracts (50 μg). **e)** Representative micrographs of SH-SY5Y cells untreated (Con) or treated for  48-h with 100 μM necrostatin- 1 (Nec), 10 μM doxorubicin (Dox) or with 100 mM necrostatin-1 plus 10 mM doxorubicin (Nec/Dox) and micrograph demonstrating cellular swelling, vacuolization and lysis of doxorubicin-treated (10 μM for 48 h) SH-SY5Y cells (bar = 50 μm) plus a histogram demonstrating significant inhibition (*) of SH-SY5Y cell-death induced by 10 μM doxorubicin (Dox) in the presence of 100 μM necrostatin-1 (Nec/Dox) at 48 h, compared to untreated controls and in the presence of 100 μM necrostatin-1 (Nec) alone, displayed as mean (± SD) percent death in three independent assays, performed in duplicate. **f** RT-PCRs of NF-YA isoform (upper panels) and GAP (lower panels) levels in SH-SY5Y cells treated for 48 h without (CON) or with TPA (10 ng/ml), retinoic acid (RA,10 μM), dibutyryl cAMP (cAMP, 0.5 mM), CoCl_2_ (150 μM), DTT (5 mM), thapsigargin (THA, 10 ng/ml) and tunicamycin (TUN, 1 μM) plus NF-YA*x* cDNA standard (NF-YAx) (upper left panel), and NF-YA isoform and GAP levels in stable TrkT3 and TrkAIII SH-SY5Y-transfectants (bottom 2 panels).

In mouse embryo RNAs, NF-YA*s* mRNA expression was predominant in stage E8.5 embryos, NF-YA*l* mRNA expression predominant in stage E9.5-E18.5 embryos and NF-YA*x* mRNA expression, confirmed by direct sequencing (data not shown), was detected in main body (M) but not head (U) or lower limbs (L) RNAs from stage E12.5, 13.5 and 14.5 embryos (Fig. 2a).

### Doxorubicin induces NF-YA*x* expression in SH-SY5Y cells

Doxorubicin (10 μM) induced NF-YA*x* mRNA expression in SH-SY5Y cells at times of maximal > 95% cell death (Fig. 2b), which in Southern blots exhibited time and concentration-dependent induction kinetics at concentrations of 5 and 10 μM, with NF-YA*x* identified by hybridization to the NF-YA exon C (Exon C) but not exon D (Exon D) probe (Fig. 2c). NF-YAx protein expression was also detected in SH-SY5Y nuclear extracts at 24 and 48 h, following Doxorubicin (10 μM) treatment (Fig. 2d). Necrostatin-1 (100 μM) significantly reduced cell death induced by 10 μM doxorubicin from 94 ± 7.5% to 74 ± 8.7% (*p* < 0.002, *n* = 12), at 48 h, (Fig. 2e) and doxorubicin-induced cell death was also characterised by vacuolation, swelling and lysis, consistent with a proportion of necroptotic cell-death (Fig. 2e). NF-YA*x* expression in SH-SY5Y cells was not induced by treatment with either DTT (5 mM), thapsigargin (10 ng/ml), tunicamycin (1 μM), TPA (10 ng/ml), dibutyryl cAMP (0.5 mM), retinoic acid (10 μM) or CoCl_2_ (150 μM) over 48 h and was not detected in either stable TrkT3 or TrkAIII SH-SY5Y-transfectants [3] (Fig. 2f).

### NF-YA*x* forms CCAAT-box binding NF-Y complexes

**Fig. 3:**
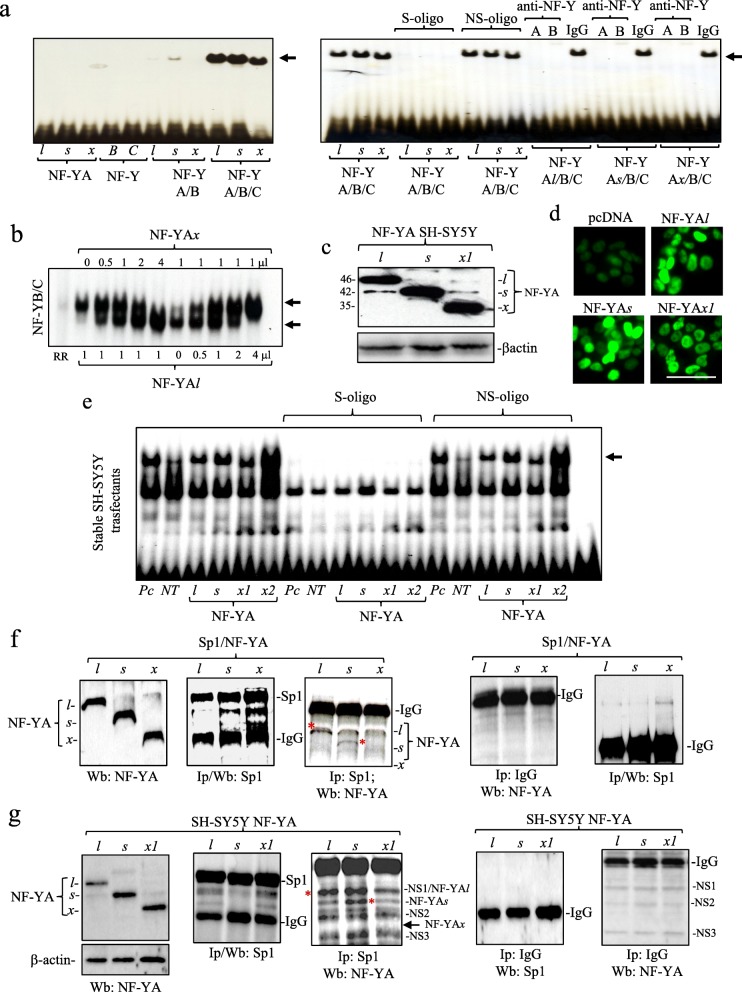
NF-YA*x* substitutes NF-YA*l* in NF-Y but does not bind Sp1. **a** EMSA demonstrating specific CCAAT-box binding NF-Y complexes (arrow) formed by in vitro translated NF-YA*l*, NF-YA*s* or NF-YA*x*, combined with NF-YB and NF-YC (left panel) plus competition EMSA showing inhibition of specific NF-Y binding complexes (arrow) in the presence of excess cold specific (S-oligo) but not non-specific (NS-oligo) oligonucleotide, and anti-NF-YB and anti-NF-YA antibodies but not pre-immune IgGs (IgG) (right panel). **b** EMSA showing changes in NF-Y electrophoretic mobility (arrows) under conditions of constant NF-YA*l* and increasing NF-YA*x* levels and vice versa*,* plus NF-YB and C, demonstrating reciprocal NF-YA*l* and NF-YA*x* substitution in NF-Y complexes*.* **c** Western blot demonstrating NF-YA*l*, NF-YA*s*, NF-YA*x* and β-actin levels in stable NF-YA*l*, NF-YA*s* and NF-YA*x1* SH-SY5Y-transfectant extracts (30 μg) (left panel) plus **d** IF demonstration of nuclear NF-YA*l*, NF-YA*s* and NF-YA*x* localization in stable NF-YA*l*, NF-YA*s* and NF-YA*x1*-SH-SY5Y transfectants (bar = 50 μm). **e** EMSA of specific CCAAT-box binding NF-Y complexes (arrow) in stable NF-YA*l*, NF-YA*s*, NF-YA*x1* and NF-YA*× 2* SH-SY5Y-transfectant nuclear extracts, with competition EMSA demonstrating specificity by inhibition with cold specific (S) but not non-specific (NS) oligonucleotides. **f** IP/Western blots showing NF-YA isoform levels in *SL2* extracts (30 μg), 72 h following co-transfection with pAc SP1 and NF-YA insect expression vectors (1st panel); Sp1 and IgG levels in Sp1 immunoprecipitates (500 μg *SL2* extracts) (2nd panel); Sp1 pull down of NF-YA*l* and NF-YA*s* (red asterisks) but not NF-YA*x* in Sp1 immunoprecipitates (500 μg *SL2* extracts) (3rd panel), and the absence of Sp1, NF-YA*l*, NF-YA*s* and NF-YA*x* in pre-immune IgG precipitates (500 μg *SL2* extracts) (4th and 5th panels). **g** IP/ Western blots of NF-YA isoform and β-actin protein levels in stable NF-YA*l*, NF-YA*s* and NF-YA*x1* SH-SY5Y*-*transfectant extracts (30 μg) (1st panel); Sp1 and IgG levels in Sp1 immunoprecipiates (500 μg extracts) (2nd panel); Sp1 pulldown of NF-YA*l* and NF-YA*s* (*) (overlapping NS1 and NS2 bands) but not NF-YA*x* (arrow) (3rd panel), and the absence of Sp1, NF-YA*l*, NF-YA*s* and NF-YA*x* but not non-specific NS1, NS2 and NS3 bands in pre-immune immunoprecipitates (500 μg of each extract) (4th and 5th panels)

In EMSAs, in vitro-translated NF-YA*l,* NF-YA*s* and NF-YA*x* all formed specific CCAAT-box binding complexes with NF-YB and NF-YC, confirmed by competition EMSAs in which excess un-labelled CCAAT-box oligonucleotide (S-oligo), anti-NF-YA and anti-NF-YB antibodies but not non-specific oligonucleotide (NS-oligo) or pre-immune IgG abrogated binding (Figs. 1f and 3a and b). EMSAs performed with constant NF-YA*l,* NF-YB and NF-YC and increasing NF-YA*x* levels (0–4 μl), or constant NF-YA*x,* NF-YB and NF-YC and increasing NF-YA*l* levels (0–4 μl), revealed reciprocal NF-YA*x/*NF-YA*l* substitution, confirmed by changes in electrophoretic mobility (Fig. 3b). EMSAs also detected specific CCAAT-box binding NF-Y complexes, containing NF-YA*l,* NF-YA*s* and NF-YA*x*, in nuclear extracts from stable NF-YA isoform SH-SY5Y-transfectants, exhibiting similar levels of nuclear-localized NF-YA*l,* NF-YA*s* and NF-YA*x* expression (Fig. 3c, d and e). NF-YA*x-*specific antibodies, however, are not available to confirm NF-YA*x* recruitment to gene promoters in vivo by chromatin immunoprecipitation assay.

### NF-YA*x* does not bind Sp1

NF-YA*l* and NF-YA*s* bind Sp1 and regulate Sp1 recruitment to promoters [11, 44]. IP/Western blots confirmed Sp1 pull-down of NF-YA*l* and NF-YA*s* but not NF-YA*x* in Sp1-negative *SL2* cells [45], co-transfected with pAc Sp1 and NF-YA*l,* NF-YA*s* and NF-YA*x* insect expression vectors (Fig. 3f). In stable SH-SY5Y-transfectants evidence for Sp1 pull down of NF-YA*l* and NF-YA*s* but not NF-YA*x* was also supported by augmented immunoreactivity overlapping non-specific (NS1 and NS2) bands, consistent with Sp1 binding of NF-YA*l* and NF-YA*s* but not NF-YA*x* (Fig. 3g).

### NF-Y*x* is cytotoxic to embryonal neural-lineage progenitors and NB cells and inhibits colony formation

**Fig. 4:**
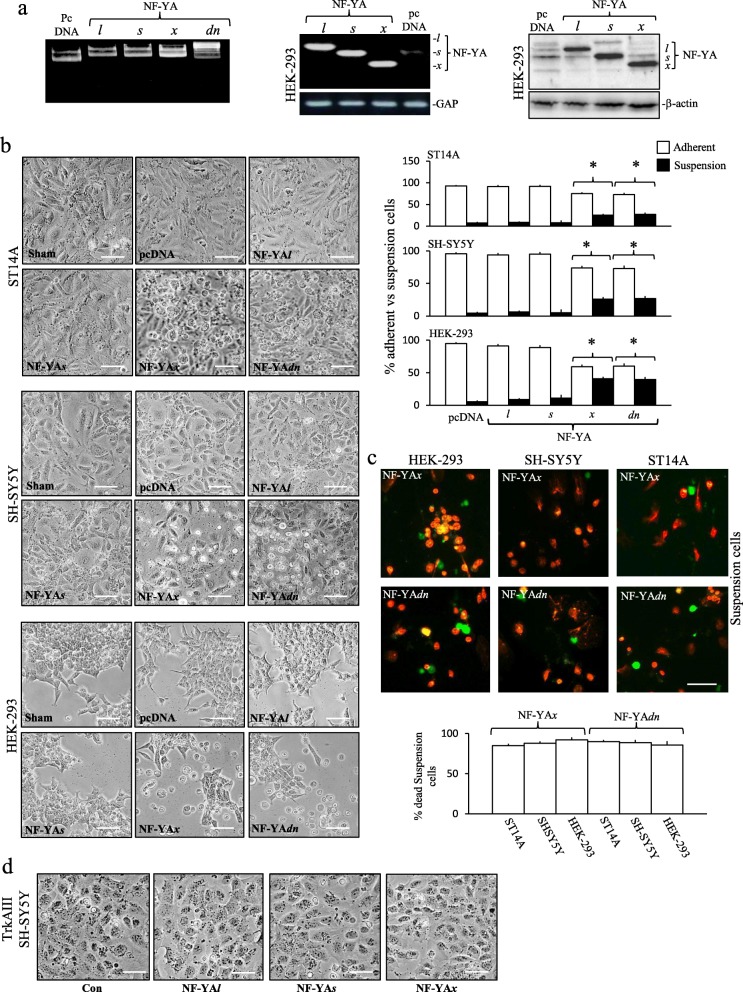
NF-YA*x* is cytotoxic. **a** Sterile purified pcDNA, NF-YA*l*, NF-YA*s*, NF-YA*x* and NF-YA*dn* plasmids used in transfections plus RT-PCR plus Western blots demonstrating NF-YA*l*, NF-YA*s*, NF-YA*x* and GAP mRNA and protein levels in HEK-293 cells, following 48 h transfection with pcDNA3.1 NF-YA*l*, NF-YA*s*, NF-YA*x* and empty pcDNA mammalian expression vectors. **b** Representative Micrographs illustrating ST14A (upper 6 panels), SH-SY5Y (middle 6 panels) and HEK-293 (lower 6 panels) cell-death, following 48 h transfection with pcDNA, NF-YA*l*, NF-YA*s*, NF-YA*x* and NF-YA*dn* expression vectors (bar = 100 μm) plus histograms demonstrating the percentage change in adherent and suspension (dead/dying) ST14A (upper), SH-SY5Y (middle) and HEK-293 (lower) cells, following 48 h transfection with pcDNA, NF-YA*l*, NF-YA*s*, NF-YA*x* and NF-YA*dn* expression vectors, results are displayed as mean (±SD) percent in three independent assays performed in duplicate (* = significant). **c** Representative AO/EtBr assays (bar = 100 μm) demonstrating HEK-293, SH-SY5Y and ST14A suspension cell death (orange/red), 48 h following transfection with NF-YA*x* and NF-YA*dn* expression vectors plus a histogram of the percentage of suspension cell-death, displayed as mean (± SD) in three independent AO/EtBr assays performed in duplicate. **d** Micrographs demonstrating lack of stable TrkAIII transfected SH-SY5Y cell-death, following 48 h transfection with either NF-YA*l,* NF-YA*s* or NF-YAx expression vectors (bar = 100 μm)

Transient 48-h transfection of ST14A, HEK-293 and SH-SY5Y with pEGFP-N1 reporter gene, resulted in transfection-efficiencies of 28.3 ± 8.2%, 55.9 ± 4.7% and 30.3 ± 6.5% (mean ± SD) (*n* = 6, for all three), respectively (not shown). Transient 48-h ST14A, HEK-293 and SH-SY5Y transfection with empty pcDNA, NF-YA*l*, NF-YA*s,* NF-YA*x* or NF-YA*dn* vectors, resulted in similar levels of NF-YA isoform expression (Fig. 4a, shown for HEK-293 cells only).

**Fig. 5:**
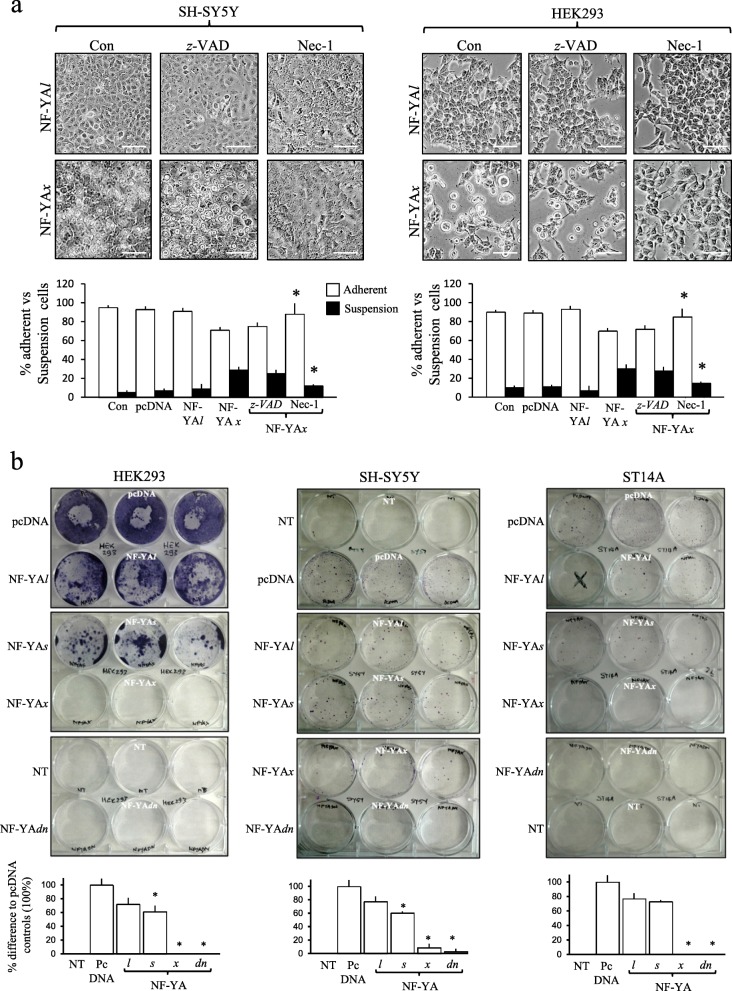
NF-YA*x* attenuates HEK-293, SH-SY5Y and ST14A colony formation. **a** Representative Micrographs (bar = 100 μm) plus histograms, demonstrating Necrostatin-1 (Nec-1) (100 μM) but not *z-*VAD*-fmk* (10 μM) inhibition of NF-YA*x-*induced SH-SY5Y and HEK-293 cell-death. Histogram data are displayed as mean (± SD) percent adherent/alive and suspension/dead cells, in three independent assays performed in duplicate (* = significant). **b** Representative colony formation assays plus histograms, demonstrating inhibition of HEK293, SH-SY5Y and ST14A colony-formation by NF-YA*x* and NF-YA*dn* compared to NF-YA*l*, NF-YA*s* and pcDNA expression vectors. Histogram data are displayed as mean (± SD) percentage difference to pcDNA-transfected controls, in three independent assays performed in triplicate (* = significant)

In ST14A cells, NF-YA*x* and NF-YA*dn* transfection significantly increased the percentage of suspension cells at 48 h, from 7.5 ± 2.25% (*n* = 6) in pcDNA, 8.5 ± 2.1% (*n* = 6) in NF-YA*l* and 8 ± 5.2% (n = 6) in NF-YA*s-*transfectants, to 25 ± 3.1% (n = 6) in NF-YA*x* and 27 ± 4.2% (n = 6) (means ± SD) in NF-YA*dn-*transfectants (*p* < 0.0001 for both NF-YA*x* and NF-YA*dn* vs controls), with 98 ± 5.5% (n = 6) of NF-YA*x*-induced suspension cells and 95 ± 7.6% (n = 6) (means ± SD) of NF-YA*dn*-induced suspension cells confirmed dead by AO/EtBr assay (Fig. 4b and c). In SH-SY5Y cells, NF-YA*x* and NF-YA*dn* also significantly increased the percentage of suspension cells at 48 h, from 4.25 ± 2.25% (n = 6) in pcDNA, 6.25 ± 2.1% (n = 6) in NF-YA*l* and 5 ± 5.2% (n = 6) in NF-YA*s-*transfectants, to 26 ± 3.1% (n = 6) in NF-YA*x* and 26.75 ± 4.2% (n = 6) in NF-YA*dn-*transfectants (means ± SD) (*p* < 0.0001 for NF-YAx and NF-YA*dn* vs controls), with 88 ± 5.5% (n = 6) of NF-YA*x*-induced suspension cells and 93 ± 5.6% (n = 6) (means ± SD) of NF-YA*dn*-induced suspension cells confirmed dead by AO/EtBr assay (Fig. 4b and c). In HEK-293 cells, NF-YA*x* and NF-YA*dn* also significantly increased the percent of suspension cells, from 5.25 ± 2.22% (n = 6) in pcDNA, 8.7 5 ± 1.9% (n = 6) in NF-YA*l* and 11.25 ± 4.8% (n = 6) in NF-YA*s-*transfectants, to 40.75 ± 3.1% (n = 6) in NF-YA*x* and 39.6 ± 4.3% (n = 6) in NF-YA*dn-*transfectants (means ± SD) (*p* < 0.0001 for NF-YA*x* and NF-YA*dn* vs controls), with 92 ± 6.5% (n = 6) of NF-YA*x*-induced suspension cells and 94 ± 7.8% (n = 6) (means ± SD) of NF-YA*dn*-induced suspension cells confirmed dead by AO/EtBr assay (Fig. 4b and c). In contrast, NF-YA*l*, NF-YA*s* or NF-YA*x* did not induce the death of SH-SY5Y cells expressing TrkAIII oncoprotein (Fig. 4d). NF-YA*x*-induced death was not detected prior to 16 h, was maximal at 48 h, was not prevented by *z-*VAD-*fmk* (20 μM) but was significantly inhibited by necrostatin-1 (100 μM) [46], from 29 ± 3.1% (*n* = 6) to 12 ± 1.6% (n = 6) in SH-SY5Y cells and from 30 ± 4.5% (n = 6) to 15 ± 1.5% (n = 6) in HEK-293 cells (means ± SD) (*P* < 0.001, for both) (Fig. 5a).

In colony formation assays, sham-transfected SH-SY5Y, HEK-293 and ST14A cells did not form colonies and pcDNA-transfected counterparts formed maximum colony numbers. Compared to respective pcDNA controls, in SH-SY5Y cells, NF-YA*l-*transfectants formed 22.6 ± 7.5% fewer colonies (*p* < 0.001, *n* = 9), NF-YA*s-*transfectants 39.5 ± 2.1% fewer colonies (*p* < 0.0001, n = 9), NF-YA*x-*transfectants 91.1 ± 5.5% fewer colonies (means ± SD) (p < 0.0001, *n* = 18) and NF-YA*dn-*transfectants did not form any colonies (p < 0.0001, n = 18). In ST14A cells, NF-YA*l* transfectants formed 23.3 ± 7.5% fewer colonies (*P* < 0.0001, n = 9), NF-YA*s* 27.3 ± 2.1% fewer colonies (P < 0.001, n = 9) whereas NF-YA*x* and NF-YA*dn-*transfectants did not form colonies (P < 0.001, n = 9 for both). In HEK-293 cells, NF-YA*l-*transfectants formed 28 ± 9.1% fewer colonies and NF-YA*s-*transfectants 38.6 ± 8.5% fewer colonies (p < 0.0001 for both, n = 9 for each), whereas NF-YA*x* and NF-YA*dn*-transfectants did not form any colonies at all (Fig. 5b).

### NF-YA*x* induces Necroptosis

**Fig. 6:**
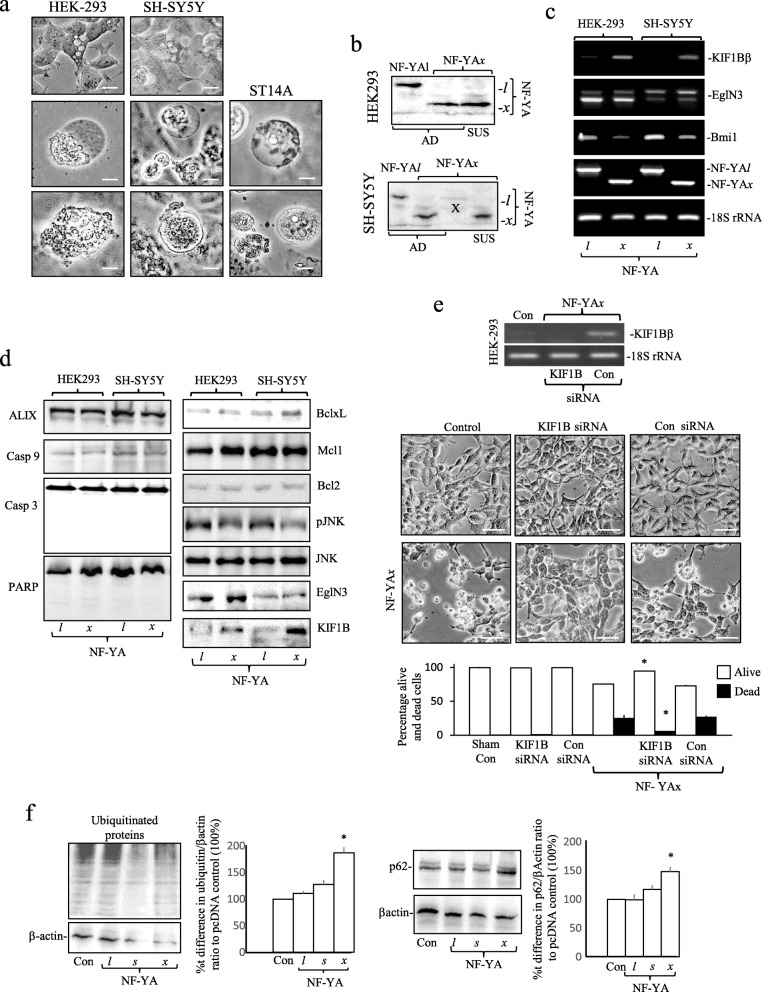
NF-YA*x* promotes KIF1Bβ-dependent necroptosis. **a** Evidence of vacuolation, swelling and necrotic-lysis in HEK-293, SH-SY5Y and ST14A cells follwoing 48 h transfection with NF-YA*x* expression vector (bar = 10 μm). **b** Western blots demonstrating the presence of both NF-YA*l* and NF-YA*x* in adherent (AD) and NF-YA*x* in dead/dying suspension (SUS) HEK-293 (upper panel) and SH-SY5Y (lower panel) extracts (20 μg), following 48 h transfection. **c** RT-PCRs showing reduced Bmi-1 expression, induction of KIF1Bβ expression but no change in EglN3 expression in HEK-293 and SH-SY5Y cells, following 24 h transfection with NF-YA*x* but not NF-YA*l* expression vector, together with NF-YA isoform and 18S rRNA levels. **d** Western blots showing stimulation of KIF1Bβ expression but no changes in ALIX, BclxL, Mcl1, Bcl2, EglN3, JNK, phosphorylated JNK levels and no cleavage of caspase 3 (Casp 3), caspase 9 (Casp 9) or PARP in HEK-293 and SH-SY5Y cells following 48 h transfection with NF-YA*x* but not NF-YA*l* expression vector (30 μg). **e** RT-PCR demonstrating KIF1B-specific (KIF1B) but not scrambled siRNA (Con) knockdown of NF-YA*x*-induced KIF1Bβ expression in HEK-293 cells compared to 18S RNA levels plus micrographs and a histogram showing prevention of NF-YA*x*-induced HEK-293 death by KIF1B-specific siRNAs (bottom 2nd panel) but not scrambled siRNAs (Con siRNA, bottom 3rd panel), compared to sham-transfected (bottom 1st panel) and siRNA-transfected controls (top 1st, 2nd and 3rd panels) (bar = 100 μm). Data are displayed as mean (± SD) percent adherent vs suspension cells in two independent experiments performed in duplicate (* = significant). **f** Western blots and histograms demonstrating significant increases in the ratio of ubiquitinated protein to β-actin in HEK-293 adherent plus suspension cell extracts (left panels and histogram) and ratio of p62 to β-actin in HEK-293 adherent plus suspension cell extracts (right panels and histogram), following 48 h transfection with NF-YA*x* compared to pcDNA, NF-YA*l* and NF-YA*s* expression vectors*.* Data are displayed as the mean (± SD) densitometric ratio in three independent experiments (* = significant)

NF-YA*x*-induced ST14A, SH-SY5Y and HEK293 cell-death was characterized by vacuolation, swelling and necrotic-lysis but not by apoptotic-body formation (Fig. 6a) or chromatin condensation (Fig. 4f). Western blots confirmed NF-YA*x* expression in dead suspension SH-SY5Y and HEK-293 cells (Fig. 6b) but did not detect caspase-3, caspase-9 or PARP-cleavage, enhanced JNK phosphorylation, reduced AIP/Alix expression or altered Bcl-xL, Bcl2, Mcl-1 and EglN3 expression (Fig. 6c and d). In both SH-SY5Y and HEK-293 cells, NF-YA*x* but not NF-YA*l* reduced Bmi1 expression, induced KIF1Bβ expression but did not alter EglN3 expression (Fig. 6c and d). In HEK-293 cells, siRNA knockdown of NF-YA*x*-induced KIF1Bβ expression significantly reduced NF-YA*x*-induced death from 24.6 ± 0.75% (*n* = 6) without siRNAs and 27.2 ± 1.2% (n = 6) with scrambled siRNAs to 5.4 ± 0.61% (n = 6) with KIF1B-specific siRNAs (means ± SD) (*p* < 0.0001 versus siRNA controls) (Fig. 6e). Western blots also detected a significant 187.08 ± 10.36% (*n* = 3) increase in ratio of ubiquitinated proteins to β-actin and significant 148.2 ± 8.6% (n = 3) increase in the ratio p62 to β-actin (means ± SD) (*p* < 0.036 for all comparisons) in extracts from HEK-293 cells transfected with NF-YA*x* compared to pcDNA, NF-YA*l* or NF-YA*s* transfected counterparts (Fig. 6f).

### NF-YA*x* selects tumorigenic, doxorubicin-resistant cancer SCs

**Fig. 7:**
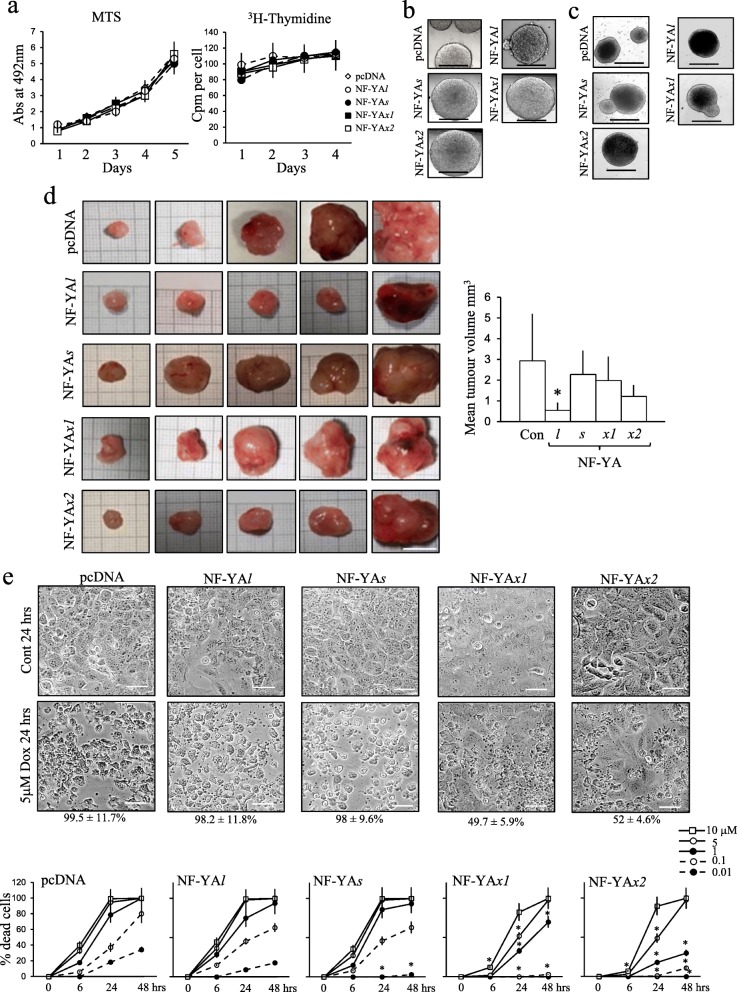
Stable NF-YA*x* transfectants are tumorigenic and doxorubicin-resistant. **a** Line graphs demonstrating similar proliferation rates in MTS (left) and ^3^H-thymidine incorporation assays by stable pcDNA, NF-YA*l*, NF-YA*s*, NF-YA*x1* and * x2* SH-SY5Y-transfectants, displayed as mean (±SD) absorbance at 429 nm for MTS assays and cpm per cell in ^3^H-thymidine incorporation assays, in three independent assays performed in duplicate. **b** Micrographs demonstrating similar neuro-sphere growth by stable pcDNA, NF-YA*l*, NF-YA*s*, NF-YA*x1* and * x2-*transfectants in neural stem cell assays (bar = 1 mm). **c)** Micrographs demonstrating similar soft agar spheroid-growth in vitro by stable pcDNA, NF-YA*l*, NF-YA*s*, NF-YA*x1* and * x2*-transfectants (bar = 1 mm). **d** Subcutaneous xenograft tumours formed by stable pcDNA, NF-YA*l*, NF-YA*s*, NF-YA*x1* and * x2*-transfectants in NGS mice (bar = 1 cm), plus histogram demonstrating mean (± SD) tumor volumes (mm^3^) for each group (* = significant). **e** Micrographs demonstrating the effect of 24 h treatment of stable pcDNA, NF-YA*l*, NF-YA*s*, NF-YA*x1* and NF-YA* x2* SH-SY5Y-transfectant with 5 μM doxorubicin (5 μM dox 24 h) compared to controls (Cont 24 h), with mean (±SD) percent death indicated below each cell line (bar = 50 μm) plus Line graphs showing differences in stable pcDNA, NF-YA*l*, NF-YA*s*, NF-YA*x1* and * x2-*stable SH-SY5Y-transfectant sensitivity to 10, 5, 1, 0.1 and 0.01 μM doxorubicin at 0, 6, 24 and 48 h, in AO/EtBr cell death assays, displayed as mean (± SD) percent death in three independent experiments performed in duplicate (* = significant)

Duplicate stable SH-SY5Y NF-YA*x-*transfectants were established and compared to stable control, NF-YA*l* and NF-YA*s*-transfectants. Stable NF-YA*l,* NF-YA*s,* NF-YA*x1* and * x2*-transfectants expressed similar levels of NF-YA*l,* NF-YA*s* and NF-YA*x* (Fig. 3c) and did not differ significantly in either proliferation or mitotic rates, assessed by MTS and thymidine-incorporation assays (Fig. 7a). NF-YA*l,* NF-YA*s* and NF-YA*x* stable transfectants all formed neuro-spheres in neural stem cell assays in vitro (Fig. 7b) and similar numbers of similar sized tumor spheroids in soft-agar tumorigenesis assays in vitro (Fig. 7c). In xenograft tumorigenesis assays in NSG mice, pcDNA, NF-YA*s,* NF-YA*x1* and * x2-*transfectants formed sub-cutaneous tumours of similar dimensions, significantly larger than tumours formed by stable NF-YA*l* -transfectants (> 2 fold smaller, *p* < 0.049 compared to all other stable-transfectants, *n* = 5 per group) (Fig. 7d). In cell death assays, doxorubicin induced similar levels of stable pcDNA, NF-YA*l* and NF-YA*s*-transfectant cell death at all concentrations at 6, 24 and 48 h. In contrast, stable NF-YA*x1* and * x2*-transfectants exhibited significantly enhanced survival at 6 but not 24 or 48 h (p < 0.0001, n = 6 for both) in the presence of 10 μM doxorubicin, at 6 and 24 but not 48 h (p < 0.0001, n = 6 for both) in the presence of 5 μM doxorubicin and at all time points in the presence of 0.01, 0.1 and 1 μM doxorubicin (p < 0.0001, n = 6 for both at all time points and doses) (Fig. 7e).

**Fig. 8:**
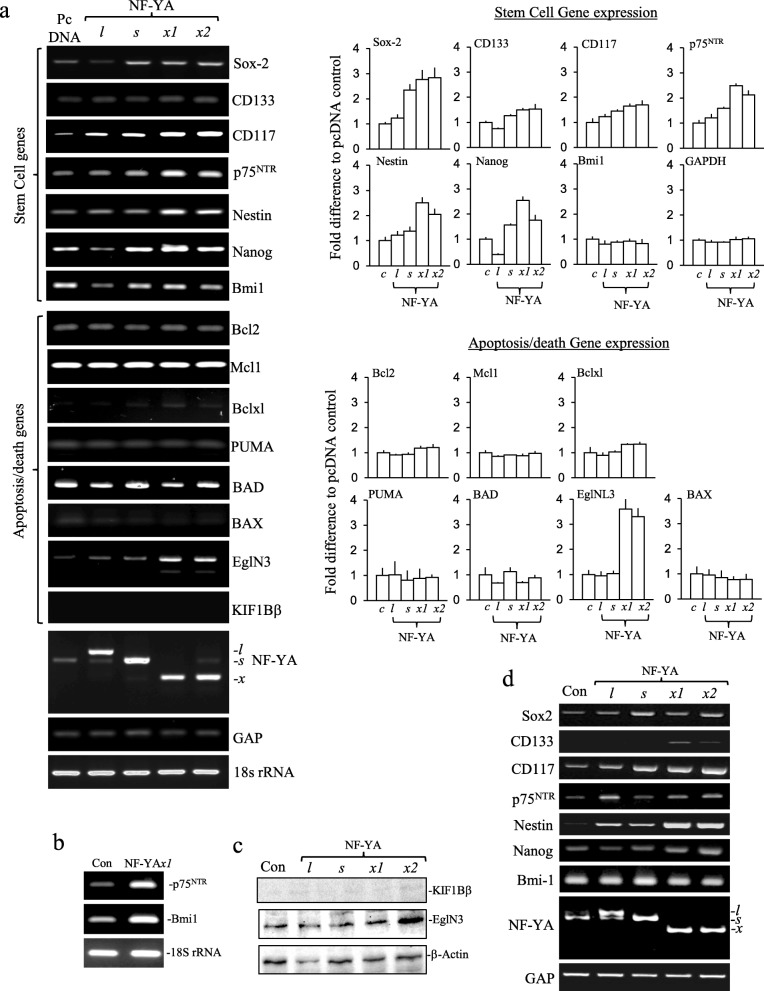
NF-YA*x* selects neuroblastoma CSCs. **a** Representative RT-PCRs and histograms demonstrating differences in stem cell-associated CD133, Sox-2, CD117, Nestin, Nanog, p75^NTR^ and Bmi1 expression and apoptosis/related EglN3, KIF1Bβ, Bcl2, Mcl1, BclxL, PUMA, BAD and BAX expression plus NF-YA*l*, NF-YA*s*, NF-YA*x,* GAP and 18S rRNA levels in stable pcDNA, NF-YA*l*, NF-YA*s*, NF-YA*x1* and * x2-*transfectants. Histogram data are displayed as mean (± SD) fold densitometric difference compared to pcDNA control transfectants, in three independent experiments performed in duplicate. **b** RT-PCR comparing p75^NTR^, Bmi1 and 18S rRNA levels in parental SH-SY5Y and stable NF-YA*x1* transfected SH-SY5Y cells. **c** Western blots demonstrating EglN3 and β-actin but not KIF1Bβ expression in pcDNA, NF-YA*l*, NF-YA*s*, NF-YA*x1* and * x2*-transfectant extracts (30 μg). **d** RT-PCRs demonstrating Bmi1, p75^NTR^, CD133, Sox-2, CD117, Nestin, Nanog, NF-YAl, NF-YAs, NF-YAx and GAP expression levels in selected xenograft tumours formed by pcDNA, NF-YA*l*, NF-YA*s*, NF-YA*x1* and NF-YA*x2*-transfectants

**Fig. 9:**
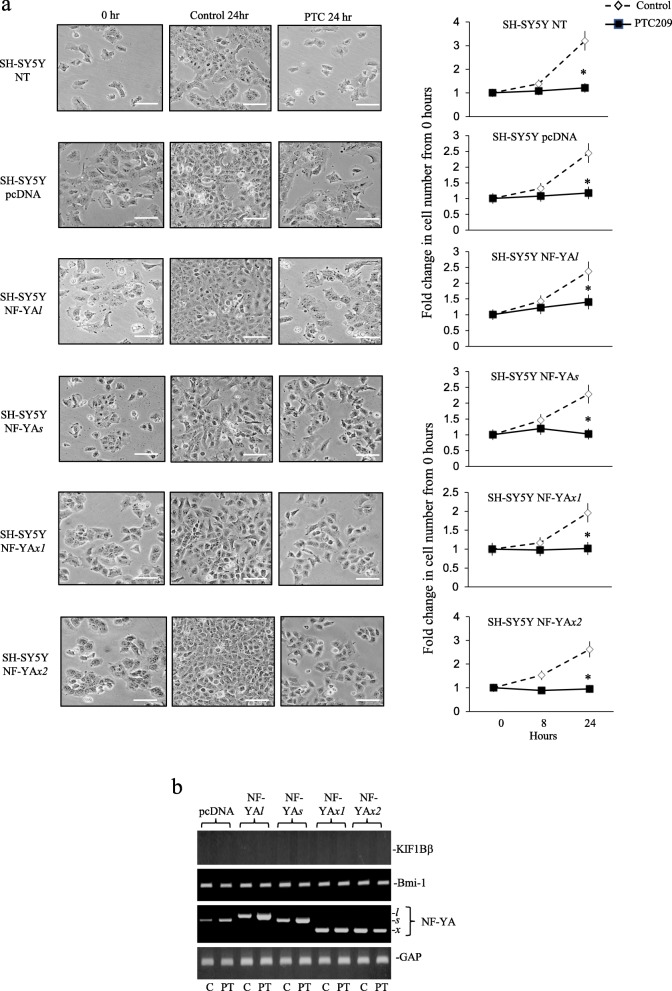
PTC-209 abrogates SH-SY5Y proliferation. **a** Phase contrast micrographs (bar = 100 μm) and histograms demonstrating abrogation of parental SH-SY5Y (NT- SH-SY5Y), stable NF-YA*l*, NF-YA*s*, NF-YA*x1* and NF-YA*x2* SH-SY5Y-transfectant proliferation, following 24 h treatment with 10 μM PTC-209. Line graph data are displayed as mean (±S.D.) fold change in cell number compared to 0-h controls, in three experiments performed in duplicate (* = significant). **b** RT-PCRs demonstrating no induction of KIF1Bβ and no reduction in Bmi-1 expression in stable pcDNA, NF-YA*l*, NF-YA*s*, NF-YA*x1* and NF-YA*x2* SH-SY5Y-transfectants, following 24 h treatment with 10 μM PTC-209

In RT-PCR assays NF-YA*x1* and * x2* transfectants exhibited higher levels of p75^NTR^, Nanog, Nestin and EglN3 expression than NF-YA*l* and NF-YA*s-*transfectants, similar levels of Sox-2, CD133 and CD117 expression to NF-YA*s-*transfectants, elevated above control and NF-YA*l*-transfectants, high-level Bmi1 expression but did not express KIF1Bβ. In contrast, NF-YA*l*-transfectants expressed lower levels of p75^NTR^ and Nanog than the other transfectants. Bcl2, Mcl1, Bcl-xL, PUMA, BAD and Bax expression levels did not differ between stable transfectants (Fig. 8a, b and c). RNAs purified from xenograft tumours exhibited a similar pattern of CSC gene expression to corresponding cell cultures (Fig. 8d)*.* PTC-209 (10 μM for 24 h) abrogated proliferation of all stable transfectants (Fig. 9a) but did not reduce Bmi1 expression nor induce KIF1Bβ mRNA expression in NF-YA*x1* or * x2-*transfectants (Fig. 9b).

## Discussion

We report a novel development and genotoxic stress-regulated alternative splice mechanism for promoting embryonic neural-lineage progenitor and NB cell-death, characterized by a switch to alternative *NF-YA* splicing and expression of a novel cytotoxic NF-YA*x* extra short-form variant*.* This novel isoform*,* originally discovered in human primary stage 2 and stage 3 NB RNAs, was the exclusive NF-YA isoform expressed at a high-level in an advanced stage 3 NB and was characterised as a novel NF-YA splice variant, exhibiting in-frame exon B, D and partial F skipping, responsible for truncating NF-YA transactivation domain sequence. NF-YA*x* readily competed with fully-spliced NF-YA*l* in CCAAT-box binding NF-Y complex formation but in contrast to NF-YA*l* and NF-YA*s* isoforms did not bind Sp1 and, therefore, represents a functional modifier of one of the more important physiological and cancer-associated transcription factors. In mouse embryos, NF-YA*x* expression coincided with the reported phase of neurotrophin-regulated KIF1Bβ-dependent sympathetic neuroblast-culling, unrestrained NF-YA*x* expression induced KIF1Bβ-dependent necroptosis in neural-lineage progenitors and NB cells and association between doxorubicin-induced NF-YA*x* expression and necroptosis in NB cells, supports a pro-necroptotic cytotoxic function for NF-YA*x* and a potential role in KIF1Bβ-dependent suppression of NB initiation, during development. On the other hand, propagation through selection of tumorigenic, doxorubicin-resistant CSC-like stable NF-YA*x* SH-SY5Y transfectants, resistant to NF-YA*x* cytotoxicity, not only helps to explain the high-level exclusive NF-YA*x* expression detected in an advanced stage 3 NB but also supports an additional potential role for NF-YA*x,* within the tumour context, in disease progression and identifies a potential mechanism for doxorubicin-induced post-therapeutic relapse, through CSC selection.

NF-YA*x* cDNA was cloned from a stage 3 NB and sequence characterised as a novel NF-YA splice variant with in-frame exons B, D and partial F sequence skipping, adding to existing NF-YA*l,* NF-YA*s* and NF-YA L2–6 variants [8, 12, 13, 17], with potential implications for NB pathogenesis and progression. NF-YA*x* expression, detected in 20% (3/15) NF-YA-positive NB RNA samples, was the exclusive high-level NF-YA mRNA isoform expressed in a stage 3 NB but was not detected in stage 1 and 4 NBs, human neonatal neural stem cells, HEK-293 human neural-lineage embryonic kidney cells and 14 human NB cell lines. Although samples were not available for NF-YA*x* protein detection in primary NBs, expression of the endogenous NF-YA*x* protein was confirmed in SH-SY5Y cells, following treatment with doxorubicin, corroborating the characterisation of NF-YA*x* cDNA as a complete, in-frame, non-mutated NF-YA splice variant that is readily translated into the NF-YA*x* protein in vitro and in vivo. Considering the relatively small number of NB samples analysed in this study, however, the possibility that NF-YA*x* mRNA expression is restricted to localized NB disseminated at most to local lymph nodes (Stages 2 and 3) [47] and may differentiate stage 3 from other disease stages, must await confirmation in a future larger NB cohort study.

NF-YA*x* mRNA was also expressed during mouse stage E12.5-E14.5 embryo development, consistent with identical exon organization of human (NCBI: NM_002505.5) and mouse (NCBI: NM_001110832.1) *NF-YA* genes. NF-YA*x* expression during stage E12.5-E14.5 embryo development coincides with the reported phase of neurotrophin-regulated EglN3/KIF1Bβ-dependent sympathetic neuroblast-culling, required for sympathetic nervous system development and suppressing NB initiation [25–28, 48–55]. Furthermore, unrestrained NF-YA*x* expression in neural-related progenitors and NB cells reduced expression of the KIF1B repressor Bmi1 [29], induced KIF1Bβ expression, promoted KIF1Bβ-dependent necroptosis and abrogated colony forming capacity in vitro. This suggests that NF-YA*x* expression during mouse embryo development may be involved in KIF1Bβ-dependent sympathetic neuroblast culling and subsequent suppression of NB initiation. In contrast, unrestrained NF-YA*x* expression did not kill NB cells expressing TrkAIII oncoprotein [3]. This bears similarity to NGF-activated TrkA protection of sympathetic neuroblast against KIF1Bβ-dependent death [26, 27] and suggests that TrkAIII, which associates with NB, may facilitate NB initiation by preventing KIF1Bβ-induced death in a manner analogous to 1p36-deletion of *KIF1B*, germline KIF1B mutations or Nmyc amplification [25–32]. NF-YA*x* induction of KIF1Bβ expression, however, did not associate with changes in EglN3 expression, suggesting that this novel death-mechanism may act down stream of EglN3 through NF-YA*x* reduction of Bmi1 expression. Whether EglN3 promotion of alternative splicing [56], extends to NF-YA*x* remains to be determined.

NF-YA*x* cytotoxicity to neural-related progenitors and NB cells was not associated with caspase 3, caspase 9 or PARP cleavage nor with changes in Bcl-2, BclXL, Mcl-1, NOXA, PUMA, BAX, BAD and AIP1/Alix expression or JNK-phosphorylation, was not inhibited by the pan-caspase inhibitor *z*-VAD-*fmk* but was characterized by cell vacuolation, swelling and necrotic-lysis and inhibited by the RIPK-1 inhibitor necrostatin-1 [46]*,* consistent with necroptosis rather than apoptosis [57, 58]. NF-YA*x* also increased the levels of ubiquitinated and p62 proteins in HEK-293 cells, suggesting reduced autophagic flux, consistent with reports that both Bmi1 inhibition and KIF1Bβ expression promote lysosomal/autophagosome uncoupling and necroptosis [59, 60], which may explain the NB suppressing function of KIF1Bβ [25, 32, 33, 54, 61, 62]. Although KIF1Bβ-induced apoptosis [32, 33, 57, 62, 63] was not detected in this study, we do not exclude potential roles for KIF1Bβ-induced ROS-mediated mitophagy or Drp1-dependent mitochondrial-fission in this death mechanism [33, 60, 64–66].

NF-YA*x* was significantly more cytotoxic to neural-related progenitor and NB cells than either NF-YA*l* or NF-YA*s*, contrasting with a previous report that unrestrained NF-YA*l* expression is highly cytotoxic and induces p53-dependent apoptosis [9]. This discrepancy can be explained by fact that p53 is compromised by SV40 large T-antigen in ST14A cells [40], by adenovirus-5 in HEK-293 cells [41, 42] and by different mechanisms in SH-SY5Y cells [67–69], suggesting that this novel NF-YA*x*-dependent necroptosis mechanism may be restricted to genotoxic-stress under p53 compromised conditions. Furthermore, the sensitivity of ST14A neural progenitors, which exhibit low-level constitutive NF-YA*x* expression, to NF-YA*x*-induced necroptosis, confirms that this mechanism depends upon predominant NF-YA*x* expression. Unrestrained NF-YA*dn* expression, which contains a DNA binding-domain mutation that prevents NF-Y binding and transcription [70], also induced HEK-293 and SH-SY5Y necroptosis. This indicates that survival can switch to necroptosis in these cell types when either NF-Y binding is prevented (i.e. with NF-YA*dn*) or when a change in NF-Y function reaches a critical level (i.e. with NF-YA*x ).* Similar necroptotic-like neuronal death has also been reported during development [25, 48, 54, 62, 71, 72]. A pro-necroptotic role for NF-YA*x* was also supported by the association between doxorubicin-induced NF-YA*x* expression and SH-SY5Y cell-death, which was also characterised by vacuolation, swelling and cell lysis and significantly inhibited by the necroptosis inhibitor necrostatin-1 [46], consistent with a percentage of doxorubicin-induced necroptosis. Confirmation of the role of NF-YA*x* in this necroptotic proportion of doxorubicin-induced death, however, must await development of reagents specific for NF-YA*x* depletion.

Doxorubicin induction of alternative NF-YAx splicing not only implicates the DNA damage-associated alternative splice mechanism [73] and a role for NF-YA*x* in the response to genotoxic stress but also identifies NF-YA*x* as a potential biomarker of response to genotoxic therapy, suggesting that NF-YA*x* expression in primary NBs could also reflect neo-adjuvant genotoxic chemotherapy [74]. Furthermore, the propagation of tumorigenic, doxorubicin-resistant, CSC-like stable NF-YA*x* SH-SY5Y transfectants, resistant to NF-YAx cytotoxicity, unveils an additional potential role for NF-YA*x* in genotoxic drug-induced, post-therapeutic relapse through CSC selection and maintenance. Moreover, the fact that NF-YA*x* expression was not induced by agents that promote ER-stress, hypoxic-stress, differentiation or malignant behaviour, suggests that this alternative splice mechanism may be relatively restricted to conditions of genotoxic stress in NB cells.

NF-YA*x* substitution of NF-YA*l* in DNA-binding NF-Y complexes and the capacity of NF-YA*l* and NF-YA*s* but not NF-YA*x* to bind Sp1 (this study, [11, 44, 75]), characterises NF-YA*x* as a potential modifier of both NF-Y and NF-Y-dependent Sp1 function. NF-YA*x* loss of Sp1 binding results from truncation of the aa 55–139 binding site for Sp1 in NF-YA*l* and NF-YA*s* to aa 55–103 in NF-YA*x* (this study, [44, 76]). This truncation may also compromise NF-Y interaction with ZHX transcriptional repressors that bind NF-YA aa’s 31–140*,* with potential to also alter NF-Y/ZHX-regulated genes expression, including MDR-1 chemotherapeutic-cytotoxicity-regulator and polo kinase-1 mitosis-regulator [77–81]. Transactivation-domain truncation may also weaken NF-Y function and interaction with other factors and the reduced size of NF-YA*x,* particularly in complexes with smaller NF-YB and NF-YC variants [82], may de-regulate transcription from promoters with precisely-spaced NF-Y-dependent transcriptional domains, e.g. ER-stress response gene promoters [83]. Although, NF-YA*x*-specific antibodies are not yet available for confirmation, by chromatin immunoprecipitation assay, that NF-YA*x* is recruited to gene promoters in vivo*,* the fact that NF-YA*x* contains an intact DNA binding domain, competes with other isoforms in NF-Y complex formation and binds double stranded inverted CCAAT-box oligonucleotides in NF-Y complexed-form in vitro with similar kinetics to other NF-YA isoforms, strongly supports this probability. Although, we are only beginning to understand how NF-YA*x* may influence transcription, transient NF-YA*x* expression reduced Bmi1 and induced KIF1Bβ expression in neural-lineage progenitors and NB cells, and stable NF-YA*x* expression was associated with enhanced p75^NTR^, SOX2, Nestin, Nanog, CD117, CD133 and EglN3 expression.

Despite the acute cytotoxicity of NF-YA*x* to SH-SY5Y cells, stable SH-SY5Y transfectants exhibiting predominant NF-YA*x* expression were eventually propagated. These transfectants did not exhibit altered Bcl2, Bcl-xL or Mcl1 expression nor constitutive KIF1Bβ expression, suggesting selection based upon KIF1Bβ repression but not Bcl2-regulated mitochondrial apoptosis. Stable NF-YAx transfectants also exhibited high constitutive Bmi1 expression, consistent with resistance to NF-YA*x*-induced KIF1Bβ-dependent cytotoxicity*.* This indicates that the SH-SY5Y cell line contains sub-populations that are sensitive and resistant to acute NF-YA*x* cytotoxicity, characterised by differences in NF-YA*x* regulation of Bmi1 and KIF1Bβ expression. The Bmi1 inhibitor PTC-209 [84], however, failed to induce KIF1Bβ expression in stable NF-YA*x*-transfectants, indicating that Bmi1 alone is not responsible for KIF1Bβ repression in the resistant subpopulations. PTC-209 did, however, abrogate the proliferation of all SH-SY5Y-transfectants, consistent with the role of Bmi1 in proliferation and reports that PTC-209 induces G1/S checkpoint cell-cycle arrest [84–89]. We are currently investigating whether KIF1B repression in stable NF-YA*x* SH-SY5Y transfectants, as a potential selection mechanism, may involve inhibition EglN3 prolyl hydroxylase activity, required for KIF1Bβ expression and regulated by oxygen and high/low-order complexing [55, 90–92] and/or changes in NB-associated NF-Y and Sp1-regulated microRNA expression [93–95].

Stable NF-YA*x* SH-SY5Y transfectants were more CSC-like than stable control, NF-YA*l* or NF-YA*s* transfectants and were characterized by higher levels of p75^NTR^, Nestin, Nanog and EglN3 expression compared to all other stable transfectants, similar levels of SOX2, CD133 and CD117 expression to NF-YA*s*-transfectants elevated over other transfectants, high-level Bmi1 expression and KIF1Bβ repression, consistent with reports that elevated Bmi1 and EglN3 expression associated with embryonic staminality [84–89, 92, 95, 96], elevated p75^NTR^ expression characterises neural crest and SH-SY5Y SCs [97–99] and KIF1Bβ-repression characterises undifferentiated aggressive NBs with 1p36 deletion or Nmyc amplification or that NBs that initiate in *KIF1Bβ* ^−/−^ mice [25, 26, 29–33, 100]. Stable NF-YA*x* SH-SY5Y transfectants were tumorigenic both in vitro and in vivo*,* confirming that acute NF-YA*x* cytotoxicity selects a tumorigenic NB CSC-like subpopulations resistant to NF-YA*x*-induced KIF1Bβ-dependent necroptosis. This provides an explanation for high-level exclusive NF-YA*x* expression in the advanced stage 3 NB and supports a role for NF-YA*x,* within the tumour context, in disease progression and suggests that doxorubicin induction of NF-YA*x* expression may also promote CSC selection in a potential mechanisms for post-therapeutic relapse, enhanced drug-resistance, increase adaptive plasticity and metastatic behaviour (this study, [101, 102]).

## Conclusions

The discovery of NF-YA*x* mRNA in primary stage 2 and 3 NBs, its expression as the exclusive isoform in an advanced stage 3 NB, expression in stage E12.5 to E14.5 mouse embryos and induction by doxorubicin in NB cells, unveils a novel NF-YA splice mechanism and variant that is both regulated by and involved in NB, development and conditions of genotoxic-stress. NF-YA*x* substitution of other isoforms in NF-Y complexes and loss of Sp1 binding capacity characterises this novel isoform as a functional modifier of one of the more important physiological and cancer-associated transcription factors. NF-YA*x* induction of KIF1Bβ-dependent embryonic neural-related progenitor and NB necroptosis, association with doxorubicin-induced necroptosis and expression in murine embryos at times corresponding to the phase of neurotrophin-regulated KIF1Bβ-dependent sympathetic neuroblast culling, supports a predominant pro-necroptotic cytotoxic function for NF-YA*x* in neural progenitors and NB cells, with potential to suppress NB initiation during development. On the other hand, propagation by selection of tumorigenic, doxorubicin-resistant CSC-like stable NF-YA*x* expressing SH-SY5Y transfectants, resistant to NF-YA*x* cytotoxicity, not only helps to explain the exclusive high-level NF-YAx expression in an advanced stage 3 primary NB but also suggests that NF-YA*x* expression within the tumour context may promote disease progression and provides a possible doxorubicin-inducible mechanism for post-therapeutic relapse, through CSC selection and maintenance, with potential to enhance survival within stressful chemotherapeutic tumour microenvironments [62, 103].

## Data Availability

The data sets used and/or analysed during this study are either included in this published article or are available from the corresponding author on reasonable request.

## Abbreviations

Bmi1: B cell-specific Moloney murine leukaemia virus integration site 1
CSC: cancer stem cell
KIF1B: kinesin family member 1B
NB: neuroblastoma
NF-YA*l*: long-form nuclear transcription factor YA
NF-YA*s*: short-form nuclear transcription factor YA
NF-YA*x*: extra short-form nuclear transcription factor YA
NSG mice: NOD severe combined immunodeficient gamma mice
Sp1: specificity protein 1
